# Understanding the benefit of metformin use in cancer treatment

**DOI:** 10.1186/1741-7015-9-33

**Published:** 2011-04-06

**Authors:** Ryan JO Dowling, Pamela J Goodwin, Vuk Stambolic

**Affiliations:** 1 Ontario Cancer Institute, University Health Network, Toronto, Ontario, Canada; 2 Mount Sinai Hospital, Toronto, Ontario, Canada

## Abstract

Biguanides have been developed for the treatment of hyperglycemia and type 2 diabetes. Recently, metformin, the most widely prescribed biguanide, has emerged as a potential anticancer agent. Epidemiological, preclinical and clinical evidence supports the use of metformin as a cancer therapeutic. The ability of metformin to lower circulating insulin may be particularly important for the treatment of cancers known to be associated with hyperinsulinemia, such as those of the breast and colon. Moreover, metformin may exhibit direct inhibitory effects on cancer cells by inhibiting mammalian target of rapamycin (mTOR) signaling and protein synthesis. The evidence supporting a role for metformin in cancer therapy and its potential molecular mechanisms of action are discussed.

## A brief history of biguanides

The biguanides metformin, phenformin and buformin are derived from the herb *Galega officinalis *(French lilac, also known as Goat's Rue or Italian Fitch) and were originally developed for the treatment of hyperglycemia and type 2 diabetes. Use of tea infused with French lilac for relief of frequent urination (polyuria) and halitosis (a sweet odor on breath), both now well known symptoms of diabetes, dates back to ancient Egypt and medieval Europe [1-3]. Work in the 1920 s identified biguanides as the active compounds from the French lilac and led to their development as therapeutics in the 1950 s [1,3,4]. While phenformin and buformin were withdrawn from the market in the 1970 s due to toxicity related to lactic acidosis, metformin (*N*',*N*'-dimethylbiguanide) remains one of the most commonly prescribed drugs, with nearly 120 million prescriptions filled yearly worldwide [5]. Metformin was approved for the treatment of hyperglycemia in Britain in 1958, Canada in 1972, and the US in 1995. In addition to its use in diabetics, metformin is also effective in the treatment of polycystic ovary syndrome and is being explored as an antiviral and anticancer agent [5-7]. Indeed, the use of biguanides in oncology was originally initiated in a series of studies targeting altered metabolism in non-diabetic cancer patients [8-10]. More recently, metformin has been associated with decreased cancer incidence and mortality in diabetic patients and the insulin-lowering effects of metformin may be integral to its anticancer properties [5,11-13]. Here, we discuss the use of metformin in oncology and its potential mechanisms of action in the inhibition of cancer.

## Discussion

### Mechanism of metformin action

At the cellular level, metformin activates AMP-activated protein kinase (AMPK), an energy sensor involved in regulating cellular metabolism that is activated by increases in the intracellular levels of AMP [14,15]. Metformin indirectly activates AMPK by disrupting complex I of the mitochondrial respiratory chain, which leads to decreased ATP synthesis and a rise in the cellular AMP:ATP ratio [16]. Increased association of AMPK with AMP under such conditions leads to stimulation of AMPK activity by three mechanisms. AMP allosterically activates AMPK and facilitates phosphorylation of its catalytic subunit on residue Thr172 by the upstream kinase liver kinase B1 (LKB1, also known as STK11), the protein product of the tumor suppressor gene mutated in the Peutz-Jeghers cancer predisposition syndrome [17]. Binding of AMP to AMPK also prevents dephosphorylation of AMPK Thr172 by protein phosphatases. Activated AMPK phosphorylates a number of downstream targets leading to stimulation of catabolic processes that generate ATP, such as fatty acid β-oxidation and glycolysis, and suppression of many of the processes dependent on ample cellular ATP supply, including gluconeogenesis, protein and fatty acid synthesis and cholesterol biosynthesis [18,19].

The mechanism of metformin action in the treatment of diabetes involves the inhibition of hepatic gluconeogenesis and the stimulation of glucose uptake in muscle [20,21]. These effects are achieved by AMPK-mediated transcriptional regulation of genes involved in gluconeogenesis in the liver and those encoding glucose transporters in the muscle, such as peroxisome proliferator-activated receptor-γ coactivator 1α (PGC-1α) and glucose transporter type 4 (GLUT4), respectively [5,22,23]. Consequently, metformin enhances insulin sensitivity and lowers fasting blood glucose and insulin in diabetics.

### Metformin and cancer

The potential for application of metformin in oncology was first recognized in retrospective epidemiological studies of diabetic patients with cancer. Numerous observational studies reported decreased cancer incidence and cancer-related mortality in diabetics receiving standard doses of metformin (1500 to 2250 mg/day in adults) [11,24-28]. For example, Evans and colleagues [11] reported a reduced risk of subsequent cancer diagnosis in diabetics receiving metformin (vs those patients not receiving the drug), with the protective effect increasing with greater metformin exposure. Additional studies examining all forms of cancer have reported reduced cancer risk in diabetics on metformin (vs no metformin treatment [24,27]) and lower cancer-related mortality in patients receiving metformin compared to those receiving other standard diabetic therapies [28]. Furthermore, a recent epidemiological study of 2,529 women with breast cancer reported higher pathologic complete response rates (pCRs; considered a surrogate for overall survival in this setting) to neoadjuvant systemic therapy in diabetic patients receiving metformin (pCR 24%) compared to diabetic patients not receiving metformin (pCR 8%) and non-diabetic patients not receiving metformin (pCR 16%)[29]. However, despite the increase in pCR, metformin did not significantly improve the estimated 3-year relapse-free survival rate in this study. Moreover, in a similar study of diabetic prostate cancer patients, metformin use was not associated with benefit [30]. Thus, further clinical research is needed to fully appreciate the impact of metformin on cancer recurrence and survival.

While the majority of evidence supporting a role for metformin in the treatment of cancer has been derived from retrospective studies involving diabetics, some prospective clinical trials have been completed in non-diabetic patients. In a recent study, low doses of metformin (250 mg/day) reduced the number of rectal aberrant crypt foci (a surrogate marker for colorectal cancer) and decreased the proliferative activity of colonic epithelium in non-diabetic patients [31]. Furthermore, interim analyses of ongoing studies involving neoadjuvant metformin treatment of newly diagnosed breast cancer patients have demonstrated that metformin is safe and well tolerated, and exhibits favorable effects on insulin metabolism and tumor cell proliferation and apoptosis [32,33].

Metformin also displays significant growth inhibitory effects in several cancer cell and mouse tumor models. In cell culture, metformin inhibits the proliferation of a range of cancer cells including breast, prostate, colon, endometrial, ovarian, and glioma [34-40]. The effects of metformin on cancer cell proliferation were associated with AMPK activation, reduced mammalian target of rapamycin (mTOR) signaling and protein synthesis, as well as a variety of other responses including decreased epidermal growth factor receptor (EGFR), Src, and mitogen-activated protein kinase (MAPK) activation, decreased expression of cyclins, and increased expression of p27. While not universally observed in all cells, metformin has been found to induce apoptosis in certain cell lines derived from endometrial cancers, glioma, and triple negative breast tumors [38,39,41].

Recent studies have demonstrated that metformin may also target cancer-initiating cells. For example, metformin inhibited the growth of a subpopulation of breast cancer cells shown to have such property in culture and reduced their ability to form tumors in mice [42] and when combined with trastuzumab, metformin reduced the cancer-initiating cell population in Her2-amplified breast cancer cells [43]. Interestingly, metformin may also be involved in regulating breast cancer-initiating cell ontogeny by transcriptionally repressing the process of epithelial to mesenchymal transition (EMT) [44]. Metformin also reduced the growth of a variety of tumor xenografts in mice including those established from breast and prostate cancer cells [36,41], and suppressed the development of breast, colon and other tumors in transgenic mice [45,46]. In addition, metformin inhibited the development of chemically induced lung tumors and preneoplastic colonic lesions in mice [47,48].

### Mechanism of action in cancer

**Figure 1:**
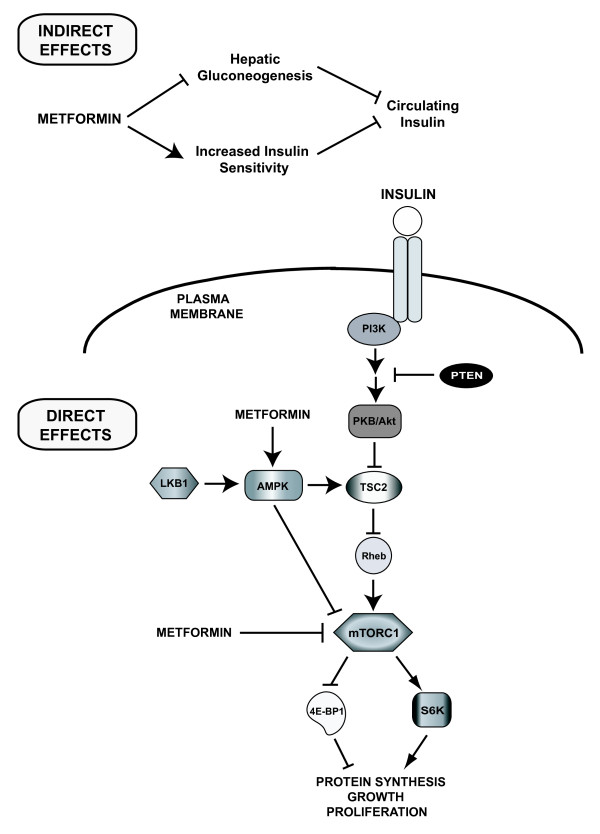
**Direct and indirect effects of metformin on cancer**. Metformin activates AMPK leading to stabilization of TSC2 and inhibition of mTORC1 signaling and protein synthesis. Metformin can also directly target mTOR independently of AMPK and TSC2. Systemically, metformin sensitizes tissues to insulin, reduces hepatic gluconeogenesis, and lowers circulating insulin levels, indirectly reducing receptor tyrosine kinase activation and PI3K signaling. AMPK = AMP-activated protein kinase; 4E-BP1 = eukaryotic initiation factor 4E-binding protein-1; LKB1 = liver kinase B1; mTORC1 = mammalian target of rapamycin complex 1; PI3K = phosphatidylinositol-3-kinase; PKB/Akt = protein kinase B; PTEN = phosphatase and tensin homologue deleted on chromosome 10; Rheb = Ras homologue enriched in brain; S6K = ribosomal protein S6 kinase; TSC2 = tuberous sclerosis complex 2.

The anticancer effects of metformin are associated with both direct (insulin- independent) and indirect (insulin-dependent) actions of the drug (Figure 1). The indirect, insulin-dependent effects of metformin are mediated by the ability of AMPK to inhibit the transcription of key gluconeogenesis genes in the liver and stimulate glucose uptake in muscle, thus reducing fasting blood glucose and insulin [1,20]. The insulin-lowering effects of metformin may play a major role in its anticancer activity since insulin has mitogenic and prosurvival effects and tumor cells often express high levels of the insulin receptor, indicating a potential sensitivity to the growth promoting effects of the hormone [49-51]. Further, obesity and high insulin levels are adverse prognostic factors for a number of cancers particularly those of the breast, prostate and colon [25,50,52-54]. Consequently, metformin may diminish the negative effects of insulin on tumor development and growth. Indeed, metformin suppressed the stimulatory effects of obesity and hyperinsulinemia on lung tumor growth in mice by improving insulin sensitivity, lowering circulating insulin, and activating AMPK signaling [13]. In addition, metformin reduced circulating insulin levels by 22% and improved insulin sensitivity by 25% in non-diabetic women with breast cancer, highlighting the insulin-lowering effects of metformin as a potential mechanism of action in the treatment of breast cancer [12].

The direct, insulin-independent effects of metformin originate from LKB1-mediated activation of AMPK and a reduction in mTOR signaling and protein synthesis in cancer cells [34] (Figure 1). AMPK impacts mTOR via phosphorylation and activation of the tumor suppressor tuberous sclerosis complex 2 (TSC2, tuberin), which negatively regulates mTOR activity [55]. mTOR is a key integrator of growth factor and nutrient signals and is a critical mediator of the phosphatidylinositol-3-kinase/protein kinase B/Akt (PI3K/PKB/Akt) signaling pathway, which is one of the most frequently deregulated molecular networks in human cancer [56,57]. Metformin-mediated AMPK activation leads to an inhibition of mTOR signaling, a reduction in phosphorylation of its major downstream effectors, the eukaryotic initiation factor 4E-binding proteins (4E-BPs) and ribosomal protein S6 kinases (S6Ks), and an inhibition of global protein synthesis and proliferation in a number of different cancer cell lines [34,35,40,58].

Some recent reports raise the possibility that metformin may mediate additional anticancer effects independently of AMPK, LKB1, and TSC2 [59,60]. Indeed, metformin reduced mTOR signaling independently of AMPK and TSC2 by inhibiting Rag GTPase-mediated activation of mTOR [59]. Paradoxically, at least in one cell model system, loss of function of LKB1 sensitized cells to the inhibitory effects of metformin under conditions of low glucose [61]. Moreover, metformin reduced hepatic gluconeogenesis by lowering hepatic energy levels in the absence of AMPK and LKB1 [60]. While these additional effects are intriguing, LKB1-dependent suppression of mTOR signaling remains the key candidate mechanism of antitumor action of metformin.

## Conclusions and future work with metformin in cancer

The clinical safety, well characterized pharmacodynamic profile, and low cost of metformin make it an ideal candidate for development as an anticancer agent. The recent convergence of epidemiologic, clinical and preclinical evidence supporting a potential anticancer effect of metformin has led to an explosion of interest in evaluating this agent in human cancer. However, a number of issues need further consideration in the development of metformin as a cancer therapy. In particular, the retrospective epidemiological studies that first identified the potential anticancer effects of metformin are difficult to confirm and contain only diabetic patient populations. While cell culture and mouse models have been integral to the characterization of the mechanism of action of metformin in the inhibition of cancer, they are artificial and rely on non-physiological doses of metformin in the presence of excess insulin and growth factors. New, more physiologically relevant *in vitro *models will be required to fully elucidate the mechanism of action of metformin (both the insulin-dependent and insulin-independent actions) and inform clinical studies. Furthermore, additional research is required to identify key patient and tumor factors that govern metformin sensitivity, which is critical for the design of clinical trials and the identification of patients best suited for metformin treatment. Current preclinical and clinical knowledge of metformin action suggest that patients exhibiting hyperinsulinemia and tumors expressing the insulin receptor, LKB1, and TSC2 would benefit most from metformin therapy, while patients with normal circulating insulin levels and tumors lacking expression of the insulin receptor, LKB1, and TSC2 would likely be unresponsive to the drug. Predicting how non-diabetic patients will respond to metformin and differentiating between its direct and indirect effects may be challenging. However, the initiation of new, focused clinical trials containing strong correlative science components will be crucial in understanding the effects of the drug on a range of cancer patients (including non-diabetic patients) and the identification of biomarkers that predict metformin benefit and response to therapy. Currently, a number of clinical trials examining the use of metformin as a cancer therapy are underway including studies in prostate, breast, endometrial and pancreatic cancer patients. In fact, the National Cancer Institute of Canada Clinical Trials Group (NCIC CTG) has initiated a large phase III clinical trial (NCIC CTG MA.32) examining the effect of metformin versus placebo in over 3,500 women with early stage breast cancer [62]. Coupled with the implementation of new preclinical models, these clinical trials will be integral to the development and effective use of metformin as a potential anticancer therapy.

## Competing interests

The authors declare that they have no competing interests.

## Authors' contributions

RD, PG and VS wrote the paper. All authors read and approved the final manuscript.

## Pre-publication history

The pre-publication history for this paper can be accessed here:

http://www.biomedcentral.com/1741-7015/9/33/prepub
